# On the dynamics of Liesegang-type pattern formation in a gaseous system

**DOI:** 10.1038/srep23402

**Published:** 2016-03-30

**Authors:** Elizeth Ramírez-Álvarez, Fernando Montoya, Thomas Buhse, Wady Rios-Herrera, José Torres-Guzmán, Marco Rivera, Gustavo Martínez-Mekler, Markus F. Müller

**Affiliations:** 1 Centro de Investigaciones en Ciencias, Universidad Autónoma del Estado de Morelos, 62209 Cuernavaca, Morelos, México; 2 Instituto de Ciencias Básicas y Aplicadas, Universidad Autónoma del Estado de Morelos, 62209 Cuernavaca, Morelos, México; 3 Centro en Investigaciones Químicas, Universidad Autónoma del Estado de Morelos, 62209 Cuernavaca, Morelos, México; 4 Instituto de Ciencias Físicas, Universidad Nacional Autónoma de México, 62210 Cuernavaca, Morelos, México; 5 Centro de Ciencias de la Complejidad, Universidad Nacional Autónoma de México, CU, DF, México; 6 Centro Internacional de Ciencias, A.C., Avenida Universidad S/N, 62131 Cuernavaca, Morelos, México

## Abstract

Liesegang pattern formations are widely spread in nature. In spite of a comparably simple experimental setup under laboratory conditions, a variety of spatio-temporal structures may arise. Presumably because of easier control of the experimental conditions, Liesegang pattern formation was mainly studied in gel systems during more than a century. Here we consider pattern formation in a gas phase, where beautiful but highly complex reaction-diffusion-convection dynamics are uncovered by means of a specific laser technique. A quantitative analysis reveals that two different, apparently independent processes, both highly correlated and synchronized across the extension of the reaction cloud, act on different time scales. Each of them imprints a different structure of salt precipitation at the tube walls.

Liesegang rings are a special type of chemical pattern formation that involves reaction- diffusion processes leading to precipitates that are not uniformly but rhythmically distributed^1^,^2^,^3^. Classical Liesegang experiments are usually performed in gel media, in which one of the reactants is homogeneously dispersed, while the other diffuses into it. Depending on the experimental set-up, a series of rings, bands, or even spherical shell precipitations can be observed^4^.

Several theories that include diffusion, reaction, nucleation, and crystal growth have been proposed to explain the Liesegang ring formation^5^,^6^,^7^,^8^. Most models are based on Ostwald’s pre-nucleation theory^9^, which explains the periodic precipitation as the consequence of a supersaturation condition necessary for nucleation that is followed by a ring deposition and a fast depletion that results in a wide zone without precipitation. Ostwald’s nucleation theory provides a physical explanation of certain scaling laws^10^,^11^,^12^,^13^, but to the best of our knowledge, none of the proposed numerical models is capable of reproducing the variety of complex patterning and peculiarities that are observed in the Liesegang experiments. In order to overcome this theoretical gap, additional experimental studies and quantitative analysis of both, the precipitated spatial structures as well as the spatio-temporal reaction dynamics, are called for.

An interesting but less studied case of Liesegang-type ring formation is the vapour-to-particle HCl/NH_3_ reaction system first reported in 1951^14^. In this particular experiment reactants are placed initially at opposite ends of an air-filled glass tube and diffuse in order to yield solid NH_4_Cl. For this case, the authors concluded that the Liesegang ring formation requires supersaturation of the individual gases, as in nucleation phenomena, and a critical cluster size necessary for the crystal growth to take place.

In a recent work, Thompson *et al.*^15^ described the outcome of a variety of experimental designs for the HCl/NH_3_ system. An amazing amount of different three -dimensional structures of the reaction cloud such as micro-tornadoes, micro-stalagmites, or micro-hurricanes has been observed, which may lead to qualitatively different precipitation patterns of varying complexity. However, in their work no further quantitative analysis was performed.

In the present contribution, we study the dynamics of the vapour-to-particle HCl/NH_3_ system using the experimental configuration for diffusing gases in the glass tube^14^. Image analysis was employed in order to observe and characterize the movement of the reaction front along the tube. Additionally we quantify possible regularities of the reaction dynamics, namely the degree of phase-synchronization within the partially diffusive, partially convective particle flow in the gas phase. This is, to the best of our knowledge, the first study where a visualization of the reaction cloud together with a quantitative analysis of its complex spatio-temporal structure is presented.

## Results

**Figure 1:**
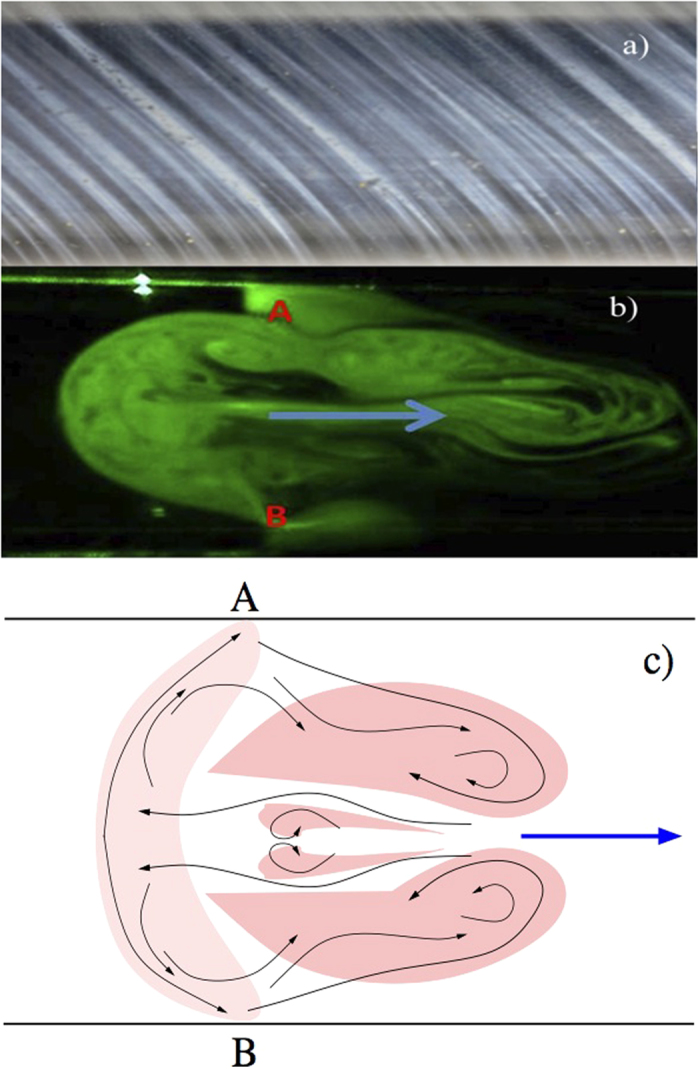
(**a**) Image of a typical spatial pattern in the HCl/NH_3_ system in the horizontal 120 × 3 cm glass tube after the reaction has settled in, and (**b)** Aerosol mushroom-shaped reaction front moving in the direction indicated by the blue arrow. Letters A and B mark the regions where the reaction cloud is in intermittent contact with the glass wall and NH_4_Cl is deposited. (**c)** Cartoon of the internal flow of micro particles within the reaction cloud. Arrows indicate mean flow direction. Regions with larger flow velocities (convection regions) are illustrated as darker pink shadows. The straight blue arrow indicates the movement direction of the whole reaction cloud.

When the chemical reaction initiates, a white fog of aerosol composed of microscopic NH_4_Cl particles appears within the inter-diffusion zone between the gaseous HCl and NH_3_. The aerosol stays at that position for a moment and subsequently the first precipitation ring formation occurs. Then it moves slowly along the tube in the direction of the HCl source producing a series of rings with different widths and separation spaces, as shown in Fig. 1a. In contrast with a vertical tube configuration, where the rings are orientated nearly perpendicular to the tube axis, the rings are inclined in the horizontal configuration used in the present study. Probably, gravity and buoyancy effects^16^ cause this inclination. In the present work we perform a quantitative observation and characterization of the time evolution of the reaction cloud itself, which complements common static structure analysis of the precipitation pattern. This strategy gives direct information on the reaction dynamics of the system and may reveal useful information about the mechanism responsible for the pattern formation. We performed three experiments obtaining quantitatively similar results. In what follows we show the outcome of one of the experiments with the exception of a summary figure at the end of the results section.

**Figure 2:**
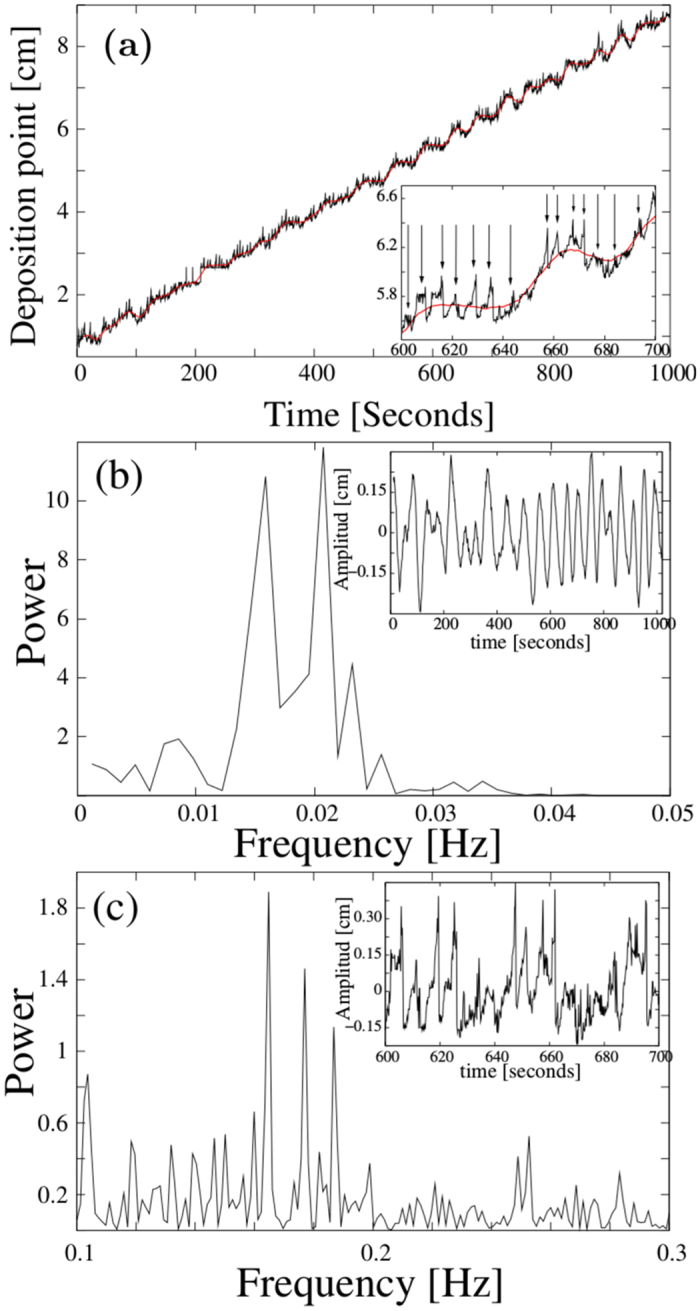
**(a)** Position of the deposition point along the tube as a function of time. The inset shows a magnification between second 600 and 700. The arrows of the inset indicate the moments where erratic bursting like excursions from the smooth part occurs. The black curve is the result of the position tracking of the deposition point. The red curve is a local average as explained in the text. **(b)** Power spectrum of the smooth part of the position-time function (red curve) shown in panel (**a**). The inset shows the time evolution of the deviations of smooth part from the linear regression. **(c)** Power spectrum of the small scale fluctuating part of the position-time function shown in panel (**a**), i.e. deviation from the smooth red line. This part is mainly characterized by burst-like changes of the position of the deposition point. The inset shows a magnification of this fluctuating part of the time series between second 600 and 700.

The reaction product NH_4_Cl aerosol is clearly visible as shown in the video image of Fig. 1b. The aerosol cloud moves slowly in direction of the HCl source (see the Supplementary Video S1). Every frame of the video was converted into light intensity matrix, where each cell corresponds to a pixel from the image. We assumed that the colour intensity at each point of the structure is directly proportional to the relative density of the NH_4_Cl in the aerosol. As described in the methods section, the tube is illuminated within a plane containing the tube axis and the orientation of the camera is perpendicular to this plane of illumination. Hence, the light does not arrive directly to the lens of the camera but is subject to multiple refraction at the micro-particles of the reaction product, which form the vapour-cloud shown in Fig. 1. Consequently, within regions of higher density of micro-particles there is a higher refraction probability. Hence one expects them to appear with a higher intensity on the photographs than those of lower vapour density. In other words, we expect that the brightness on a photograph is a direct measure for the vapour density within the tube. Figure 2c shows a cartoon of the reaction cloud internal dynamics of the main flow of NH_4_Cl micro particles.

One observes in the Supplementary Video S1 that the turbulent right hand part of the cloud produces almost periodically a vapour stream that gets inserted into the central region and pushes the temporarily stable half-moon structure on the left. This provokes a stronger bending of the structure separating its outer regions from the tube wall (at positions A and B of Fig. 1b,c). During this phase, the deposition rate of salt material at the tube wall is noticeably reduced. Instead, the vapour flows from the lateral regions A and B of Fig. 1b,c towards the turbulent region of the reaction cloud and the whole cloud moves forward to the right (as indicated by the blue arrow in Fig. 1b,c.). Thereafter, the bending of the half-moon structure decreases and contact at the tube wall is re-established with the onset of a higher deposition rate of salt material, but now at a new position on the glass tube.

### Study of the velocity profile

In order to follow the position of the mushroom-shaped aerosol along the tube, we track the deposition point marked with the letter A and B in Fig. 1b,c. The movement of point A is shown in Video S1 of the Supplementary Material marked as a pink coloured square.

Tracking the deposition region during the experiment we can reconstruct the position of the reaction cloud as a function of time. This result is displayed in Fig. 2a. (cf. corresponding figure on page 149 of reference^15^). On the average, the displacement of the reaction front occurs with a constant mean velocity of about 7.92 × 10^−3^ cm/s, determined by a linear regression of the data. This linear behaviour is modulated by a step-like function. The smooth part of the displacement is depicted by the red curve shown in Fig. 2a, which is a local running average. By taking the difference between the local average and its linear regression, i.e. a straight line with a slope equal to the average velocity, the smooth component is extracted. The result of this difference is shown in the inset of Fig. 2b.

According to this result, the reaction cloud advances in thrusts of varying strengths and dwell times. It remains at a position for a certain while, or even moves slightly backwards, until it thrusts forward along the tube. This process occurs with a mean frequency of about *f≤0.02* *Hz*, clearly visible as a broad double peak in the power spectrum shown in Fig. 2b. This quasi-periodic characteristic is superposed by bundles of narrow spikes, which may occur erratically at any instant (see the inset of Fig. 2a) and resembles a burst-like behaviour. Taking the difference between the position function (black curve of Fig. 2a) and its local average (red curve of Fig. 2a) separates this faster activity. A section of the resulting time series is shown in the inset of Fig. 2c. The burst frequency varies strongly between 0.1 and 0.25 Hz during the duration of the experiment, showing three main maxima between 0.16 and 0.19 Hz clearly visible in the power spectrum shown in Fig. 2c. Hence, the movement of the reaction cloud can be characterized by a mean constant velocity superposed by two processes operating on well separated time scales.

**Figure 3:**
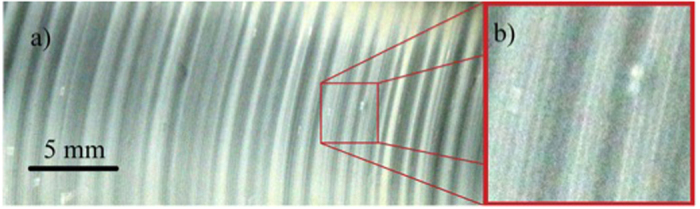
(**a**) Picture of the ring pattern captured with a Canon camera EOS Rebel T4i with a macro canon lens EF-S 6 mm. (**b**) In the red square to the right, zoom into three rings of the picture showing the fine structure of narrow bands, which occur within the broad bright regions, as well as within dark regions, where less of reaction product is precipitated.

A closer inspection of the photographs of the glass tube reveals that also the precipitation pattern shows structures on two different (spatial) scales (Fig. 3). Zones where a larger amount of the reaction product is deposited can be seen as broad, brightly shining bands. Their intensity and widths, as well as the widths and intensity of the dark regions vary in an irregular fashion along the tube. This pattern is superimposed by a fine structure of narrow rings. They occur equally in brightly shining as well as dark regions with alternating intensity. We relate the broad band structure with the stepwise advance of the reaction cloud (Fig. 2a), viz. the slow process with a main frequency below 0.02 Hz, while we associate the narrow rings with the faster bursting activity. Eye revision of Video S1 provided in the Supplemental Material supports this hypothesis.

### Time evolution of the spatial intensity pattern

**Figure 4:**
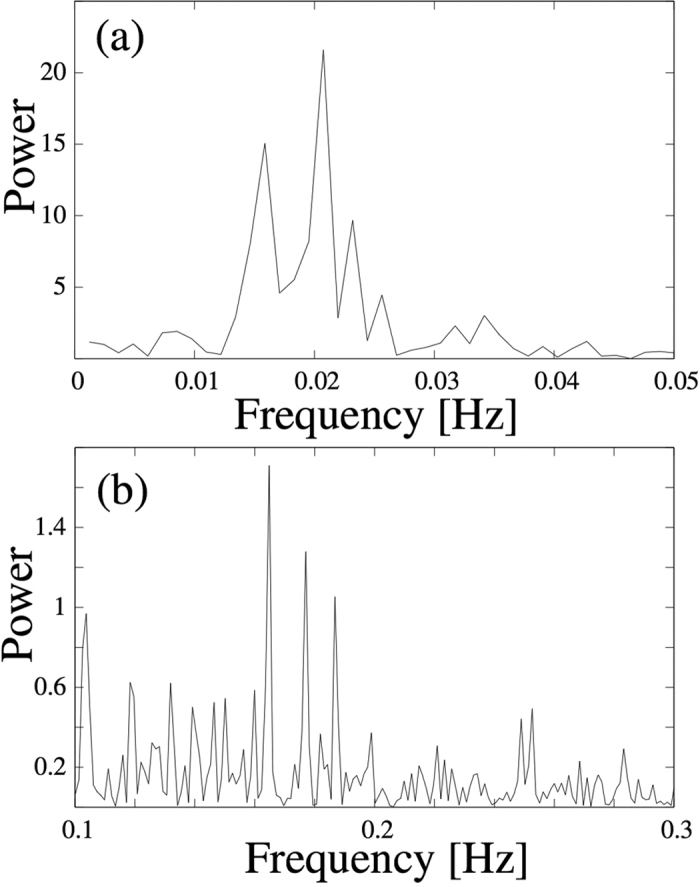
Average power spectrum of the intensity profile of the deposition point for slow frequencies (panel **(a)**) and a faster frequency range (panel **(b)**).

To this end, we analysed the time series derived from the intensity of the deposition point and hence, according to the argumentation given above, the density of the micro-particle cloud by estimating its power spectrum. The results are displayed in Fig. 4 separately for the low and fast frequency range, corresponding to the smooth dynamics and the bursting behaviour extracted from the position function of the deposition point. Comparing the results obtained from the intensity with those of the spatial variation of the deposition point, one observes a striking similarity between the power spectra. Although the total power of the curve shown in Fig. 4a is notably larger than the one presented in Fig. 2b, relative changes are very similar. All maxima and minima appear at almost identical frequencies for both, the low and the faster frequency range. For instance, the broad double maximum below 0.2 Hz is equally present, as well as the three prominent peaks, between 0.16 and 0.19 Hz. Even details of the fine structure of the curves shown in Fig. 2b,c are reproduced in the graphs shown in Fig. 4a,b. The localization of the deposition point is therefore highly correlated to its intensity in the photographs and therefore also to the vapour density of micro salt particles.

This result fits the scenario sketched in the discussion of the velocity profile (Figs 2 and 3), where we argued that during the dwells between the quasi-periodic thrusts of the whole cloud a broader region of precipitation appears, while the bursting of the deposition point generates the fine structure of intense narrow bands. This is more likely if during these episodes the density of the reaction cloud within the region of particle deposition is higher, so that more material can be deposited at the tube walls. Hence, the brightness of the deposition region as a function of time should display the same characteristics as the time evolution of its spatial position. Therefore, the similarity of the results displayed in Fig. 4a,b should not be unexpected.

### Spatial interrelations within the reaction cloud

In order to support the findings elucidated in the last sections, we examine now the cloud dynamics from a different point of view; namely, we study the time evolution of spatial correlations between different parts of the reaction cloud. For this purpose we shift each frame of Video S1 so that the pixel related to the deposition region was kept at a fixed position within the figure (see Video S2 of the supplementary material), i.e. we change to the reference frame of the moving reaction cloud. For each image a mesh of N = 30 × 32 rectangles of size 20 × 10 pixels was superimposed and the mean intensity of each rectangle was estimated (we have also chosen other sizes of the rectangles obtaining similar results). A band pass filter (0.08–0.25 Hz) was used to correct the illumination effect described in the methods section. This allows us to study the correlation dynamics of the cloud exclusively in the frequency band associated with the fast process.

**Figure 5:**
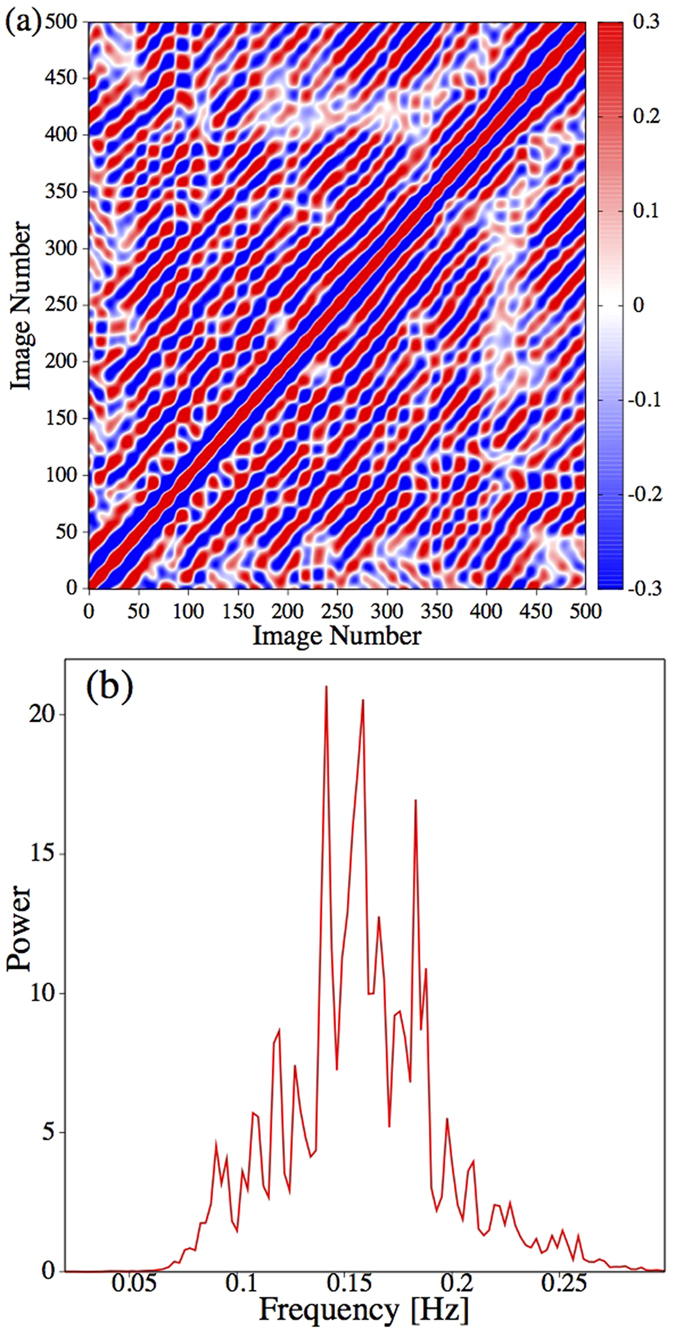
(**a**) Correlation matrix quantifying the similarity of 500 intensity vectors extracted from the images of Video S1 as explained in the text. The time span of 500 images corresponds to 100 seconds. (**b)** Average power spectrum of the time correlations between spatial intensity patterns presented by the complete correlation matrix *C*_*ij*_ estimated over the full recording of about 1000 seconds. The width of the pronounced peak coincides with the frequency range of the fast process discussed in the text.

Once the above was accomplished, we ordered the *n* intensity values at time “*i*” in a data vector *x*_*i*_(*n*). Then we calculated the Pearson correlation between all time pairs. In this way, we obtain a matrix *C*_*ij*_, defined explicitly in the methods section, that reflects similarities of the spatial intensity pattern measured at different times “*i*” and “*j*”. A time interval of these results is shown in Fig. 5a.

The most interesting features of matrix *C*_*ij*_ are the diagonal red and blue lines covering the whole matrix. The spatial intensity pattern is repeated up to a certain precision in an almost periodic manner such that epochs of time correlation (red lines) and anti-correlation (blue lines) alternate. This quasi-periodicity of the spatio-temporal correlation structure is somewhat unexpected, given that the power spectra of the time series (extracted from intensities or position changes) show several sharp peaks of varying amplitude. However, the time evolution of spatial correlations seems to be remarkably regular, indicating alternating but well-established phase relationships of the spatial intensity distribution over time. On the average, each row of *C*_*ij*_ contains 17 maxima, i.e. every 30 images, similar spatial intensity pattern appear. This is equivalent to a frequency of about 0.16–0.17 Hz, which is just in the centre of the fast frequency range and coincides approximately with dominant frequencies of the power spectra shown in Figs 2c and 4b. This observation is confirmed by averaging the power spectra estimated for each row of *C*_*ij*_ (Fig. 5b). Notice that despite the averaging procedure both width and structure of the peak zone coincide with those of Figs 2c and 4b.

However, the magnitude of the correlation coefficients of matrix *C*_*ij*_ varies between ± 0.3, which is not a remarkably high value. Therefore, the main significance of the result presented in Fig. 5a consists essentially in the ordered diagonal pattern, which is spread over the whole matrix though it is not that much reflected in the magnitude of the matrix elements. An interesting question that comes to mind at this point is whether the comparably small but systematically alternating correlation values arise because only a small and localised fraction of the density pattern of the spatial extended cloud repeats itself almost exactly in a quasi-periodic fashion, while the rest of the cloud evolves in an irregular and turbulent manner; or alternatively, whether the density of an ample region of the cloud shows quasi-periodic dynamics but is superimposed by a considerable noise background caused by turbulent fluctuations of the vapour density. This aspect will be addressed with more detail in the next section.

### Spatial activity pattern and synchronization analysis

We start with a visualization of the dynamics of the reaction cloud itself, viz. the spatial intensity distribution as a function of time. This is done separately for the fast and slow frequency domains identified above.

**Figure 6:**
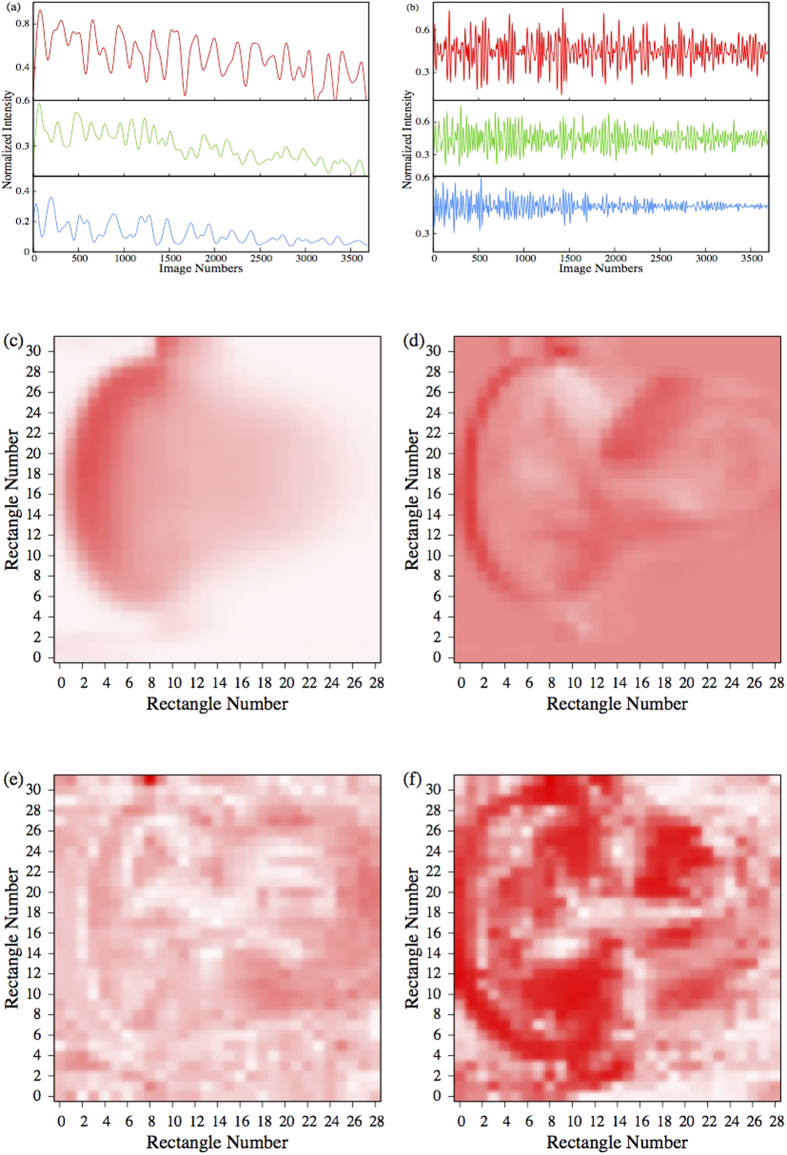
**(a)** Normalized intensity time series of the slow frequency band selected from the rectangle at the deposition point (upper panel, red curve), a rectangle from the left boundary of the half-moon structure of the reaction cloud (middle panel, green line) and a rectangle selected from the far right of the convection tail region (lower panel, blue curve). (**b)** Normalized intensity time series for the fast frequency band. Color-coding and rectangle selection is the same as above. (**c)** Average of the images of Video S2, showing the activity of the slow frequency band. (**d)** Snap shot of the activity of the fast frequency band. (**e)** Mean phase coherence between the rectangle containing the deposition point and the remaining ones, estimated for the slow frequency band. **(f)** Snap shot of the mean phase coherence between the deposition rectangle and the remaining ones for the fast frequency band. The color scale of figure (**e,f**) varies between zero (white) and one (intense red).

The results for slow and fast frequency components are shown in the Videos S2 and S3 respectively. In both cases one observes a global decrease of the intensity in: (a) the vertical direction of the figures and (b) towards the end of the video. These artificial effects are caused for case (a) by the fact that the laser source was positioned above the tube and for case (b) because the reaction cloud moves outside the optical range of the camera. In Fig. 6 we provide an overall presentation of our visualizations. First notice that for all the plotted signals, the mean amplitude and intensity variance decrease as time evolves.

The Video S2 reveals very slow oscillations superposed by an offset of nearly zero frequency. The main magnitude of this slow activity is mostly concentrated on the far left side of the reaction cloud, namely on the outermost left border of the half-moon structure. The intensities measured within the tail of the right hand side are noticeably diminished. In Fig. 6a the time series extracted from rectangles at the deposition point, the left boundary of the half-moon structure and the convective tail are plotted. In Fig. 6c. an average over the images of Video S2 is presented. Regions with large amplitude activity, marked as deep red zones, are mainly located on the left border of the reaction cloud.

The situation changes when faster frequencies are considered. Figure 6b shows the behaviour of the intensity time series for the same rectangles chosen in Fig. 6a, while Fig. 6d provides a snapshot of the activity pattern of the fast frequency band. For these frequencies large amplitude activity is distributed over the whole extension of the reaction cloud.

Video S3 clearly exhibits a spatially extended pulsating activity pattern within the contours of the reaction cloud shown in previous figures. This is in contrast with the perception in Video S1 of an irregular behaviour for which, despite some rhythmic overtones, one is never under the impression that the dynamics within the reaction cloud is characterized by an ordered state of vibration. In Video S3 one observes that a particular oscillation pattern settles in for a while before it evolves to another oscillatory mode and may reappear after a sequence of different states of vibration is passed through. In any case, the visual inspection of the videos reaffirms that the evolution of the intensity oscillations of the fast frequency band is by no means an expression of a stochastic, uncorrelated dynamical state.

In order to quantify possible interrelationships between different regions of the reaction cloud we estimate the mean phase coherence^17^ (MPC) presented in the methods section. This has the advantage over linear correlation measures of detecting phase locking independently from the average phase shift between signals. We calculated the mean phase coherence *R*_1*i*_ between the intensity signal extracted for the rectangle containing the deposition point and the intensities of all the other rectangles. The results are shown in Fig. 6e,f as well as in the Video S4 for the fast activity.

Although the large amplitude slow activity was mainly confined in a comparably small region at the left border of the reaction cloud (Fig. 6c), phase synchronization within this frequency band, determined via MPC, is prominent over the whole spatial range of the aerosol distribution. While the magnitude of the slow activity alternates notably between different parts of the cloud, there is (in a statistical sense) an almost constant phase relationship between the deposition region and all other parts. Surprisingly, phase synchronization seems to be even slightly more pronounced between the deposition point and the right convection region than with the half-moon area on the left, which is mainly characterized by large amplitude slow oscillations.

In contrast to the above finding, pronounced phase synchronization within the fast frequency domain between the rectangle of the deposition region and the outermost left border is observed. Areas with a considerably high synchronization level are distributed over the whole region of the reaction cloud, although for this frequency band a more irregular behaviour could be expected. This observation is confirmed by Video S4, which shows the time evolution of the mean phase coherence between the deposition point and the remaining rectangles. The video reveals fluctuations on small spatial scales but an amazingly stable synchronization pattern in a global sense. Irregular fluctuations are mainly visible on small spatial scales of the order of one rectangle, while on spatial scales of the order of the cloud-dimension, the reaction-diffusion-convection dynamics of the reaction product is extremely stable in time and fixed phase relationships are established.

**Figure 7:**
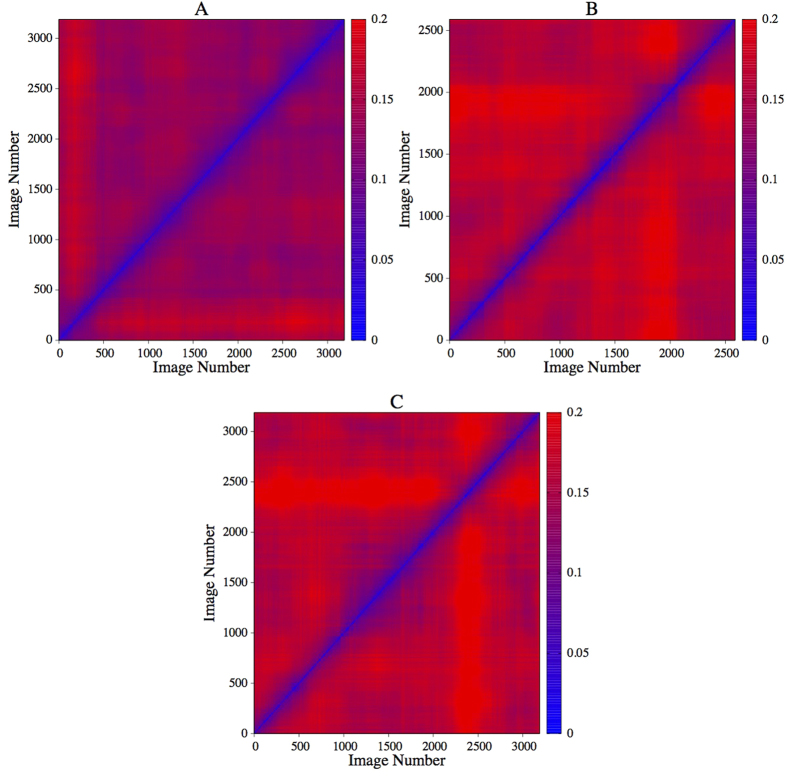
Difference matrices as defined by equation (6) for the three conducted experiments. Rows and columns of each matrix indicate the image number for which the mean phase coherence matrices *R*_1*j*_ have been estimated. The time span of 3000 images corresponds to 600 seconds. Panel (**A**) shows the case of the experiment documented throughout the paper, the matrices shown in panel (**B,C**) resume the results obtained for the remaining two experiments.

The difference matrix *D*_*kl*_ defined in the methods section, depicted in Fig. 7, quantifies the similarity between the spatial synchronization patterns *R*_1*i*_ estimated at different times denoted by “*k*” and “*l*”. The matrix *D*_*kl*_ provides a compact view of the whole time evolution shown in Video S4.

Figure 7 shows that on the average the differences between matrix elements of the mean phase coherences *R*_1*i*_ taken at different times are small for all three experiments. Panel A of Fig. 7 displays the difference matrix derived from the experiment reported so far. Panel B and C refer to further experiments performed. The median values of the three matrices are 0.13, 0.16 and 016, respectively. Lower values of the 95% significance interval are given by 0.08, 0.96 and 0.1, while the upper values are estimated as 0.17, 0.2 and 0.21, respectively. Hence, differences are in all cases surprising small, with tiny fluctuations around its median value. This means that the overall similarity between the synchronization patterns *R*_1*i*_ estimated at different times is high. However, within the small variation of the matrix elements of *D*_*kl*_ one observes an alternation of periods with higher and smaller similarity in all three cases. We attribute this behaviour to the continuous changes of modes of vibration of the intensity pattern within the fast frequency range as illustrated by Video S3. Overall, the intensity pattern shows at any time ordered spatially extended oscillations, which switch eventually between different modes. Furthermore, by the visual inspection of Video S3 one is under the impression that the continuous change of the oscillation pattern occurs in cycles, such that the same (or similar) pattern is re-established after some while. This fits the numerical result presented in Fig. 7, where the intuitive impression of the video is quantitatively substantiated by the peculiar structure of the matrix *D*_*kl*_: periods of higher and lower similarity alternate. This observation holds for the three experiments, which is documented by Fig. 7. The difference matrices display in all three cases a striking pattern of horizontal and vertical stripes, indicating a recurrent similarity of the ever changing oscillation pattern.

These results underline, that the somewhat small values of the correlation matrix (Fig. 5) are not because of a high similarity within a spatially restricted region of an otherwise disordered and uncorrelated reaction cloud. Instead we disclose that its anatomy is given by amazing ordered oscillating structures (on two different time scales), which cover the whole extension of the reaction cloud.

Finally, the question arises as to whether fast and slow activities are coupled or if they constitute independent processes acting on different time scales. Therefore we checked for possible cross frequency coupling like e.g. if the phase of the low frequency oscillations modulates the amplitude in the fast frequency range. Nevertheless, we did not find any significant relationship of such type when analysing the intensity profile of the rectangle of the deposition point. However, this does not imply that such interrelations do not exist. It may be that they are present but too weak and that the influence of non-stationarities and noise hamper the retrieval any convincing evidence.

## Discussion

We presented the experimental and theoretical analysis of a reaction-diffusion-convection system, where two gases diffuse toward each other within a glass tube producing a reaction product that deposits at the tube wall. As a result of this process, a sequence of irregular rings of deposited salt is observed (Figs 1 and 3). These are irregular in the sense of erratically spaced rings of apparently random width and intensity. Additionally, the whole structure is embedded in an amorphous background of randomly deposited micro-particles, which appears in Figs 1 and 3 as a kind of grey shadow. A closer inspection of this precipitation pattern reveals that the alternating zones of deposited material are superimposed by a fine structure of intense narrow rings, generated by a very localized deposition of NH_4_Cl (Fig. 3). This observation suggests that the salt deposition occurs via two different, seemingly independent or weakly coupled processes.

Another observation worthwhile emphasizing is the spatially extended complex structure of the reaction cloud with regions of higher and lower mean velocity of micro particles as shown in Fig. 1 and visualized in Video S1. Our study is mainly focussed on the quantitative analysis of the time evolution of the reaction cloud.

Because of the presence of the convection region, which has evidently a major influence on the internal dynamics of the reaction cloud, the term “Liesegang pattern” as a description of the observed structure should be taken with caution. Classical Liesegang patterns occur in gel systems^1^,^2^,^3^ where no convection takes place. Therefore we suspect that the generating mechanism of the observed pattern is somehow more complex than that of classical experiments. Hence, we adopted the terminology “Liesegang-type” pattern for the phenomena observed in the present study.

When tracking the position of the deposition point along the tube as a function of time one observes a constant mean velocity profile, which is modulated by a smooth step function and an irregular bursting expressed by accumulations of sharp spikes (see Fig. 2a). The smooth modulation has been attributed to the generation of the broader regions with alternating average concentration of precipitated material, while the irregular bursting has been related to the formation of the fine structure of intense narrow rings.

Considering that the mean velocity of the reaction front is about 8 × 10^−3^ cm/s and the period of the slow frequency is approximately 50 seconds, the mean distance between two adjacent broader region maxima should be of the order of 4 mm. Visual inspection of the precipitation pattern corroborates this estimate. The fast frequency band, on the other hand, is about 8 times faster. Hence, the appearance of about 8 rings of the superposed structure within each broader precipitation zone is expected and confirmed by visual inspection of the precipitation pattern.

Large part of the present work was concerned with the study of the time variation of light intensities, measured at different sites of a cross section of the reaction cloud. Here the main assumption is that higher/lower aerosol concentrations lead to a higher/lower probability of light refraction and consequently to higher/lower intensity regions of the photographs. Hence, the light intensity is assumed to be a direct measure for the aerosol concentration. This was validated by means of a power spectrum analysis of the intensity measured at the rectangle containing the deposition point. We observed a strikingly good coincidence of the spectrogram derived from the intensity measurement (Fig. 4) and that one calculated from the position-time function (Fig. 2). All major maxima appear at the same frequencies and furthermore the fine structure of the whole power spectra coincides surprisingly well. This means that high light intensity values at the deposition point are measured precisely both, during the dwell times of the step function shown in Fig. 2a, as well as during the burst periods, viz. during the periods where salt material is deposited at the tube walls and when highest aerosol concentration within the deposition region is required. Consequently, this finding supports the assumption that the light intensity of the photographs is a direct measure for aerosol concentration.

By studying the spatial properties of the reaction cloud we encountered an almost periodic repetition of the spatial correlation pattern as documented by Fig. 5a. The main frequency of the structure clearly visible in the correlation matrix coincides nicely with the range of the fast frequency band identified by the univariate analysis of position and intensity of the deposition point. Hence, one might suspect that there is a pronounced interrelation between the local dynamics of the deposition point, in particular the accentuated bursting activity, and the extended spatial dynamics of the whole reaction cloud.

In order to investigate this issue in more detail we visualized the activity pattern of the two frequency bands previously identified. We observed that the slow activity is mainly confined within the very left part of the reaction cloud (Fig. 6c) while the fast activity covers its whole extension (Fig. 6d). Nevertheless, for both frequency ranges comparably high values for the mean phase coherence between the rectangle containing the deposition point and the remaining ones were determined, which were indicative of pronounced phase locking. This finding is highlighted by Videos S3 and S4, which show the time evolution of the fast activity and the corresponding mean phase coherence values respectively. In particular Video S3 reveals that the fast activity encounters at almost all instances an ordered spatial oscillation. In fact, the precise oscillation mode changes frequently while the experiment evolves, traversing a sequence of different oscillation patterns. This sequence seems to be repeated in a kind of cycles.

However, Fig. 7 reveals that the phase locking pattern is quite stable over time. Changes are mainly effective on a small spatial scale of the order of the extension of only one rectangle, while on larger scales the synchronization pattern keeps almost constant (see Video S4 of the Supplementary Material). Hence, although the spatial oscillation pattern changes continuously (see Video S3 and Fig. 7) the synchronization properties stay (almost) constant.

Furthermore, we suspect that there is some coupling between the activity of the fast and slow frequency band. This suspicion is corroborated by the fact that the synchronization profile obtained independently for the two frequency ranges covers the same area, although we could not find any significant indication for this hypothesis in terms of e.g. phase-amplitude coupling.

Additional matters that deserve special attention, for which we have only disclosed qualitative trends, are moisture, diameter and temperature dependences of the pattern formation. With regard to the effect of water vapour we confirmed the finding of^18^ that under moisture free conditions no Liesegang rings were formed. Concerning tube diameter, we found that as it increases a regular ring spatial distribution^18^ gives way to the type of patterns discussed in this paper. This might be related to the advent of a non-negligible convection contribution to the reaction-diffusion process.

The temperature issue is fundamental for a proper understanding of the phenomenon under study. Both external condition temperature and internal temperature distribution are important. With regard to the environment temperature we found that the structure here under study disappears below 17 °C and is replaced by a homogeneous salt-layer deposited at the tube.

Overall, we believe that a finely orchestrated multifactor phenomenon, involving temperature and concentration gradients of diverse origin and their interrelations, are the key elements for the understanding of our observations^19^. We are dealing with an open system in which the exothermic nature of the chemical reaction provokes both temperature and concentration gradients. Since the system is principally governed by chemical reaction, diffusive fluxes and convective motions (the latter admissible for sufficiently large tube diameters), some aspects could probably be rationalised in terms of a reaction-diffusion-convection system^18^,^19^,^20^. The consideration of convection is a new feature in studies of gas-phase Liesegang-type pattern formation. Further experimentation, such as measurement of the internal temperature distribution, particle tracking, quantitative determination of cloud geometry-flux interrelations, amongst others, is required in order to disentangle in detail the question of how reaction, diffusion and convection originate and generate the observed precipitation pattern

Summing up, our main results are: the visualization of the propagating pattern forming phenomenon, its identification with a reaction-diffusion-convection process, the discovery of a fast deposition mode previously undetected, the stability along the evolution of the reaction cloud of the synchronization between the activity at the deposition region and all the remaining regions of the reaction cloud.

## Methods

### Experimental set-up

All experiments were performed in sealed and horizontally mounted glass tubes with an internal diameter of 3 cm and a length of 120 cm. Cotton balls (0.15 g) soaked with 1ml of aqueous HCl (37%) and 1ml of aqueous NH_3_ (28–32%) were placed each at one end of the tube. To roughly control the position where the first ring formation takes place and considering the difference between the diffusion constants of the gases, *D*HCl = 0.16 cm^2^/s and *D*NH_3_ = 0.22 cm^2^/s at 1 atm and 25 °C^21^,^22^, HCl was set in first. The tube was maintained at room temperature of ~25 °C.

**Figure 8:**
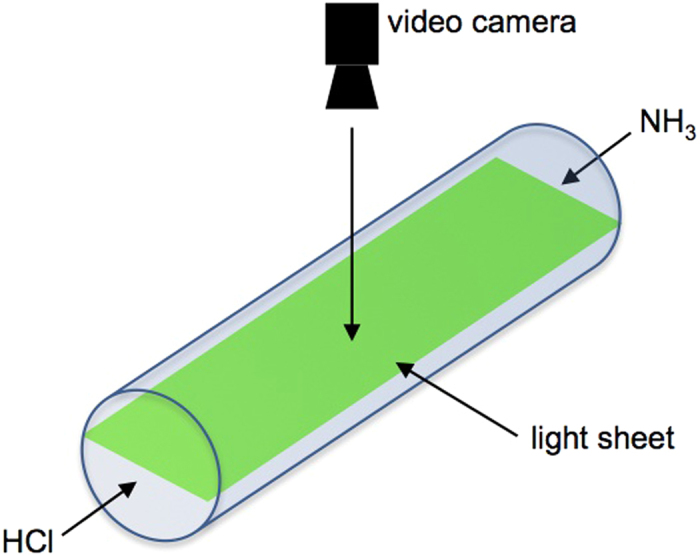
Experimental set-up consisting of a horizontally mounted glass tube with a laser light-sheet beam illuminating the axial plane of the tube and a video camera placed at the top to record the aerosol dynamics.

As indicated in Fig. 8, a green laser light-sheet was used to illuminate the tube along the length direction. The light-sheet was placed just in the middle of the tube circumference. The observed precipitation pattern was not influenced by local heating of the laser beam as confirmed by other experiments without laser illumination that showed similar results. A video camera was placed at the upper part of the tube at a distance of approximately 5  cm in order to record the dynamics of the aerosol propagation within that plane. Video S1 (see Supplementary Material) illustrates the typical dynamics of the reaction cloud. It was recorded with a *Logitech* webcam (*HD Pro Webcam C920*) with a resolution of 1280 × 720 pixels and 5 frames per second. Three experiments were carried out with this configuration. Though the here shown data was derived from one of the experiments, similar results hold for the other two experiments as documented by Fig. 7.

### Time correlation

For each image a mesh of N = 30 × 32 rectangles of size 20 × 10 pixels was superimposed and the mean intensity of each rectangle was estimated. The mean intensity values of the N rectangles were then assigned as components of a data vector *x*_*i*_(*n*) at time index ‘*i*’. Then the zero-lag correlation was determined between all time pairs of these data vectors:


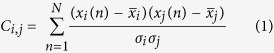


In this formula, the sum goes over the *N* rectangles and the indices ‘*i*’ and ‘*j*’ refer to different time points. 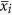 and *σ*_*i*_ are the mean and standard deviation estimated over the *N* components of the intensity vector at time frame ‘*i*’.

### Mean Phase Coherence

Here we follow an analytical signal approach^23^,^24^,^25^ using the Hilbert Transform


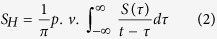


where *p.v.* indicates the principal value for the determination of the instantaneous phase of

the signal *S*(*t*) defined as


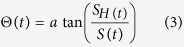


Once the instantaneous phases are estimated a possible phase locking between two signals *S*_*i*_(*t*) and *S*_*j*_(*t*) can be quantified by


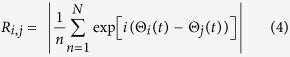


A reliable estimation of the mean phase coherence is only possible for mono-component narrow band signals. A comprehensive overview about the applicability of techniques based on the estimation of instantaneous frequencies can be found in ref. 26. Therefore, we performed such estimates separately for the slow and fast processes based on the data derived from the light intensity measurements.

For the slow frequency band a segment of 4096 data points was studied. The segment length for the *R*_*ij*_ estimation of the fast frequency band was 256 data points or equivalently 256 photographs, which was then shifted with a maximal overlap across the recording. In both cases about 10% of the data points at each border were disregarded for the estimation of the instantaneous phases in order to diminish the influence of finite size effects.

### Time evolution of light intensities

Here we focus on the intensities extracted for each rectangle from the images of Video S1. To this end the images were set in the reference frame of the moving reaction cloud. Then, for each rectangle the average intensity was measured as a function of time. The ensuing time series were then filtered in two frequency bands, the slow band between 0 and 0.05 Hz and the fast frequency band between 0.08 and 0.25 Hz. For the filtering procedure a 4^th^ order Butterworth filter^27^ was applied in forward and backward direction in order to minimize possible shifting of the signal phases. However, filtering causes, in particular for the fast frequency bands that the time evolution of the filtered intensities oscillate around zero. High pass filtering eliminates slow frequency contributions and thereby may produce a possible offset. As a result the filtered signals can take positive as well as negative values, which is counterintuitive if intensities are under consideration. Therefore, we added to the filtered intensity values the minimal value of the signals derived from all rectangles. Thereafter we divided each of the 960 signals by the highest absolute maximum of all of them. Hence the intensity values of all time series vary between zero and one.

### Quantification of the time stability of the spatial synchronization pattern

In order to quantify the time stability of the synchronization pattern within the fast frequency band, we estimated an average difference matrix in the following way:


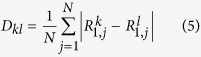


Here 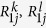 denote the mean phase coherence between the rectangle of the deposition point and rectangle ‘*i*’, estimated at times ‘*k*’ and ‘*l* ’ respectively while ‘*N* ’ denotes the number of possible pairs of rectangles, which is in the present case N = 928.

## Additional Information

**How to cite this article**: Ramírez-Álvarez, E. *et al.* On the dynamics of Liesegang-type pattern formation in a gaseous system. *Sci. Rep.* **6**, 23402; doi: 10.1038/srep23402 (2016).

## Supplementary Material

Supplementary Information

Supplementary Video 1

Supplementary Video 2

Supplementary Video 3

Supplementary Video 4
