# Supplementary material for: On the dynamics of Liesegang-type pattern formation in a gaseous system
Source: Sci Rep. 2016 Mar 30;6:23402. doi: 10.1038/srep23402 (PMC4812250; doi:10.1038/srep23402)
Supplement: Supplementary Information [file srep23402-s1.doc]

On the dynamics of Liesegang-type pattern formation in a gaseous system

Elizeth Ramírez Álvarez1,%,& , Fernando Montoya2,**,& , Thomas Buhse3 , Wady Alexander Rios

Herrera2 , José Concepción Torres Gúzman1 , Marco Rivera1,# , Gustavo Martínez-Mekler4,5,6,# , Markus

F. Müller1,5,6,#,*

1 Centro de Investigaciones en Ciencias, Universidad Autónoma del Estado de Morelos, 62209 Cuernavaca,

Morelos, México.

2 Instituto de Ciencias Básicas y Aplicadas, Universidad Autónoma del Estado de Morelos, 62209

Cuernavaca, Morelos, México.

3 Centro en Investigaciones Químicas, Universidad Autónoma del Estado de Morelos, 62209 Cuernavaca,

Morelos, México.

4 Instituto de Ciencias Físicas, Universidad Nacional Autónoma de México, 62210 Cuernavaca, Morelos,

México.

5 Centro de Ciencias de la Complejidad, Universidad Nacional Autónoma de México, CU, DF, México.

6 Centro Internacional de Ciencias, A.C., Avenida Universidad S/N, 62131 Cuernavaca, Morelos, México.

* Corresponding author: muellerm@uaem.mx

% Presently at Nonequilibrium Chemical Physics, Physik-Department, TU-München, James-Franck-Str. 1,

85748 Garching bei München, Germany.

** Presently at Instituto de Biotecnología, Universidad Nacional Autónoma de México, Av. Universidad

2001, Cuernavaca, Morelos 62210, México.

&These authors contributed equally to this work.

# These authors contributed equally to this work.

In the supplementary material we provide four videos in order to visualize different aspects of the dynamics of the reaction cloud:

V**ideo S1:** Time evolution of the reaction cloud, visualised as a longitudinal cut of the glass tube by means of the laser technique described in the methods section.

**Video S2:** Time evolution of the slow component of the intensity time series as described in the text and summarised in Figure 6.a,c

**Video S3:** Time evolution of the fast component of the intensity time series as described in the text, documented as a snapshot in Figure 6.d

**Video S4:** Time evolution of the mean phase coherence between the rectangle containing the deposition point and the rest of the cloud, estimated for the fast component. A snapshot is shown in Figure 6.f
